# Frequency-specific network topologies in the resting human brain

**DOI:** 10.3389/fnhum.2014.01022

**Published:** 2014-12-22

**Authors:** Shuntaro Sasai, Fumitaka Homae, Hama Watanabe, Akihiro T. Sasaki, Hiroki C. Tanabe, Norihiro Sadato, Gentaro Taga

**Affiliations:** ^1^Graduate School of Education, University of TokyoTokyo, Japan; ^2^Department of Psychiatry, University of Wisconsin - MadisonMadison, WI, USA; ^3^Department of Language Sciences, Tokyo Metropolitan UniversityTokyo, Japan; ^4^Pathophysiological and Health Science Team, Imaging Application Group, Division of Bio-function Dynamics Imaging, RIKEN Center for Life Science TechnologiesKobe, Japan; ^5^Department of Physiology, Osaka City University Graduate School of MedicineOsaka, Japan; ^6^Graduate School of Environmental Studies, Nagoya UniversityNagoya, Japan; ^7^Division of Cerebral Integration, Department of Cerebral Research, National Institute for Physiological SciencesOkazaki, Japan; ^8^Department of Physiological Sciences, SOKENDAI (The Graduate University for Advanced Studies)Okazaki, Japan

**Keywords:** resting-state fMRI, frequency-dependency, network analysis, functional connectivity, rich-club connectivity, community, integration, segregation

## Abstract

A community is a set of nodes with dense inter-connections, while there are sparse connections between different communities. A hub is a highly connected node with high centrality. It has been shown that both “communities” and “hubs” exist simultaneously in the brain's functional connectivity network (FCN), as estimated by correlations among low-frequency spontaneous fluctuations in functional magnetic resonance imaging (fMRI) signal changes (0.01–0.10 Hz). This indicates that the brain has a spatial organization that promotes both segregation and integration of information. Here, we demonstrate that frequency-specific network topologies that characterize segregation and integration also exist within this frequency range. In investigating the coherence spectrum among 87 brain regions, we found that two frequency bands, 0.01–0.03 Hz (very low frequency [VLF] band) and 0.07–0.09 Hz (low frequency [LF] band), mainly contributed to functional connectivity. Comparing graph theoretical indices for the VLF and LF bands revealed that the network in the former had a higher capacity for information segregation between identified communities than the latter. Hubs in the VLF band were mainly located within the anterior cingulate cortices, whereas those in the LF band were located in the posterior cingulate cortices and thalamus. Thus, depending on the timescale of brain activity, at least two distinct network topologies contributed to information segregation and integration. This suggests that the brain intrinsically has timescale-dependent functional organizations.

## Introduction

Functional connectivity indicates statistical dependency with the activity between brain regions, implying that they share information. Since similar spatial characteristics of functional connectivity have been demonstrated during both task execution and rest, the brain might not be a stimulus-responsive organ. Rather, it might function through the intrinsic activity constraints (Biswal et al., 1995; Fox and Raichle, 2007). Functional magnetic resonance imaging (fMRI) has been used to investigate functional connectivity across the whole brain; related findings have provided a fundamental view of the brain's spatial organization that simultaneously achieves information segregation and integration (Sporns, 2013). The brain has a characteristic network structure that promotes independence between clusters of regions, as well as enhances inter-dependence across all areas. Furthermore, fMRI signals contain multiple time-scale components; the coexistence of fMRI signals fluctuating at several time scales has been shown (Zuo et al., 2010; Baria et al., 2011; He, 2011). However, the relevance of these time-scale components to the spatial architecture of functional connectivity is unclear. Therefore, the current study investigated frequency specificity of the brain's functional connectivity profile that contributes to information segregation and integration.

Spatial organization of spontaneous brain activity has been studied from the viewpoint of graph theory. Based on this theory a functional connectivity network (FCN) is constructed by viewing functional connectivity as an edge, and each brain region as a node. The FCN also has sub-network structures consisting of densely interconnected regions called “modules” or “communities.” Recent studies have provided evidence suggesting that different communities are less correlated to each other and have own differentiated functions (Dosenbach et al., 2007, 2010; Power et al., 2011; Fornito et al., 2012; Spreng et al., 2013). Conversely, highly connected central regions are called “hubs” (Achard et al., 2006; van den Heuvel et al., 2008b; Buckner et al., 2009; Tomasi and Volkow, 2011a,b). Hubs are thought to be important for integration in FCN by bridging between different communities via those called “connectors” (Sporns et al., 2007; Hagmann et al., 2008; Meunier et al., 2009; Power et al., 2013), and by forming a higher-order structure, referred to as “rich-club” with dense interconnections (Crossley et al., 2013; Grayson et al., 2014). The presence of these network structures indicates that FCN has a topology enabling the network to concurrently segregate and integrate information.

It has been demonstrated that fMRI signals contributing to functional connectivity exhibit “low-frequency fluctuations” within 0.01–0.10 Hz (Cordes et al., 2001). Therefore, most fMRI studies on network-level organization of functional connectivity have focused on synchronized low-frequency fluctuations of fMRI signal changes (0.01–0.10 Hz) in the resting brain (Fox and Raichle, 2007). However, a computational study demonstrated that topological features of functional connectivity could vary with the timescale of brain activity without changing the underlying structural connections (Honey et al., 2007). Indeed, frequency-specific characteristics exist in correlations with hemodynamic fluctuation within this low-frequency range, and differ depending on the particular combination of brain regions (Wu et al., 2008; Chang and Glover, 2010; Sasai et al., 2011). Furthermore, an fMRI study has demonstrated that some brain regions not only show event-related activity occurring at typical timescales for hemodynamic responses to a single event (0.05–0.10 Hz), but also display signal increases that are sustained for the duration of a task block (Dosenbach et al., 2006). These studies suggest that FCN derived by from fMRI could have frequency-specific topologies that potentially have different functional relevance with specific information structures; however, to our knowledge, no previous report exists regarding his issue. Thus, the present study investigated the relationship between different frequency components of spontaneous fMRI signal fluctuations and network structures responsible for information segregation and integration in FCN.

## Materials and methods

### Participants

A total of 28 healthy adults (15 men and 13 women; age range, 22–44 years) participated in this study. The protocol was approved by the ethical committee of the National Institute for Physiological Sciences, Okazaki, Japan. Informed consent was obtained from all participants prior to taking part in the study.

### Data acquisition

All MRI data used in the current study were obtained during simultaneous near-infrared spectroscopy (NIRS). All participants were instructed to remain awake with their eyes closed during data acquisition, and confirmed after the process that they had not fallen asleep. These data were similar to those used in a previous study (Sasai et al., 2012). The following sections briefly introduce the method used to obtain MRI and NIRS data in the previous study.

#### MRI

Structural and functional volumes were acquired using a 3-Tesla MR scanner (Allegra; Siemens). First, a time-series of 610 volumes was acquired for each session using a T2^*^-weighted gradient-echo echo-planar imaging (EPI) sequence. Each volume consisted of 34 slices, each of which was 3.5-mm thick with a 17% gap. The time interval between two successive acquisitions of the same slice (TR) was 2000 ms, with a flip angle (FA) of 76°, and an echo time (TE) of 30 ms. The field of view (FoV) was 192 × 192 mm, and the in-plane matrix size was 64 × 64 pixels. Additionally, to acquire a fine structural whole-brain image, magnetization-prepared rapid-acquisition gradient-echo (MP-RAGE) images were obtained (*TR* = 2500 ms; *TE* = 4.38 ms; *FA* = 8°; FoV = 230 × 230 mm; one slab; number of slices per slab = 192; voxel dimensions = 0.9 × 0.9 × 1.0 mm).

#### NIRS

We used a near-infrared optical topography instrument (ETG-4000; Hitachi Medical Corporation, Tokyo, Japan) to measure the time series of spontaneous changes in oxygenated and deoxygenated hemoglobin concentrations with a 0.1-s time resolution. The instrument generated two wavelengths of near-infrared light (695 and 830 nm). We evaluated relative changes in the oxygenated and deoxygenated hemoglobin signals from an arbitrary baseline (set to 0) at the beginning of the measurement period based on the Lambert–Beer law. The unit used to measure these values was molar concentration multiplied by length (mM·mm). The distance between the incident and the detection fibers was 3 cm. The eight emitters and eight detectors were plugged into a holder, to which vitamin tablets were attached to identify the positions of NIRS channels in MRI images, and were arranged into two 1 × 8 arrays, resulting in 14 measurement channels. Arrays were positioned over the bilateral frontal, temporal, and occipital regions by referring to the international 10–20 System of Electrode Placement. NIRS data were simultaneously obtained with fMRI imaging for all participants, with each participant lying supine in an MRI scanner.

### fMRI preprocessing

Functional MR volumes were motion-corrected and slice-timing-corrected using the SPM8 package (Wellcome Department of Imaging Neuroscience, London, UK). fMRI data sets were spatially smoothed with a 5-mm full-width-at-half-maximum Gaussian blur, and were normalized to the MNI space using DARTEL in SPM8. fMRI data sets are generally contaminated with noise, including fluctuations due to scanner instabilities, subject motion, and respiration and cardiac effects, resulting in coherent signal fluctuations across the brain (e.g., global signals). In many studies, these contaminating signals are estimated by utilizing fMRI data-inherent information, and removed using a general linear model (GLM) technique (Fox et al., 2005). However, the regression of global signal has been shown to introduce spurious anti-correlations (Murphy et al., 2009; Anderson et al., 2011). Anderson et al. (2011) have proposed an alternate method for avoiding this bias in correlation estimation; it uses an optimally phase-shifted waveform extracted from soft tissues of the face and calvarium, as well as regressors obtained from subject motion parameters, white matter, ventricles, and physiological waveforms, and is termed phase-shifted soft-tissue correction [PSTCor]. Based on the method proposed by Anderson et al. (2011), we previously used a modified version of the PSTCor that only employed fMRI inherent information, and observed no anti-correlation (Sasai et al., 2012). The present study applied this modified method of PSTCor to eliminate noise.

### ROI selection

We refer to network of the brain as “FCN,” while we call its sub-structure like default mode system as a “functional system” hereafter. It has been suggested that spontaneous brain activity is organized into two widespread functional systems in terms of the activity profiles recruited by cognitively demanding tasks: “task-positive systems” and “task-negative systems (Fox et al., 2005).” Although several studies have consistently reported activation within regions including the dorsal anterior cingulate cortex, frontal insula, lateral prefrontal cortex, and lateral parietal cortex during attention and working memory tasks (Menon et al., 2001; Curtis and D'Esposito, 2003; Kerns et al., 2004; Ridderinkhof et al., 2004; Fox et al., 2005), reduced activity in in brain regions such as the medial prefrontal cortex, angular gyrus, and posterior cingulate cortex have also been observed during these tasks (Gusnard et al., 2001; Raichle et al., 2001; Fox et al., 2005). Recently, it has been shown that the task-positive system consists of at least two different sets of brain regions in terms of its functions: central executive and saliency systems (Seeley et al., 2007; Menon and Uddin, 2010). Furthermore, Dosenbach et al. (2006) demonstrated that the task-positive system is composed of multiple sub-systems, including the fronto-parietal system overlapping with central executive systems, and the cingulo-opercular system overlapping with the saliency system, whereas the task-negative system is composed of a single system (the default mode system). In order to investigate the existence of frequency-specific topology in a large-scale FCN, we selected the following three functional systems, which included hub regions from both task-positive and task-negative systems: the default mode system (DMS), the fronto-parietal task control system (FPS), and the cingulo-opercular task control system (COS) (Power et al., 2011). Dosenbach et al. (2010) had identified coordinates corresponding to these functional systems by conducting a meta-analysis of the relevant literature. We used the reported coordinates to extract the time series corresponding to the functional systems. These ROIs were located in areas of the cerebral cortex and sub-cortical regions. The total number of ROIs was 87. Table 1 summarizes the MNI coordinates, originally assigned functional systems, and names of automated anatomical labeling (AAL) of the ROIs (Tzourio-Mazoyer et al., 2002).

**Table 1:** **List of coordinations of ROIs**.

**No**.	**MNI**			**Dosenbach**	**Network**	**AAL**	**Assigned community**	
	***x***	***y***	***z***				**VLF**	**LF**
1	6	64	3	vmPFC	Default	Frontal_Sup_Medial_R	1	1
2	0	51	32	mPFC	Default	Frontal_Sup_Medial_L	1	1
3	−25	51	27	aPFC	Default	Frontal_Mid_L	1	2
4	9	51	16	vmPFC	Default	Cingulum_Ant_R	1	1
5	−6	50	−1	vmPFC	Default	Cingulum_Ant_L	1	1
6	−11	45	17	vmPFC	Default	Frontal_Sup_Medial_L	1	1
7	8	42	−5	vmPFC	Default	Cingulum_Ant_R	3	3
8	9	39	20	ACC	Default	Cingulum_Ant_R	3	3
9	46	39	−15	vlPFC	Default	Frontal_Inf_Orb_R	1	2
10	23	33	47	Sup frontal	Default	Frontal_Sup_R	1	2
11	−16	29	54	Sup frontal	Default	Frontal_Sup_L	1	1
12	52	−15	−13	Inf temporal	Default	Temporal_Mid_R	1	1
13	−59	−25	−15	Inf temporal	Default	Temporal_Mid_L	1	1
14	1	−26	31	Post-cingulate	Default	Cingulum_Mid_R	2	3
15	28	−37	−15	Fusiform	Default	Fusiform_R	1	1
16	−3	−38	45	Precuneus	Default	Cingulum_Mid_L	1	1
17	−8	−41	3	Post-ingulate	Default	Calcarine_L	1	1
18	−61	−41	−2	Inf temporal	Default	Temporal_Mid_L	1	1
19	−28	−42	−11	Occipital	Default	Lingual_L	1	1
20	−5	−43	25	Post-cungulate	Default	Cingulum_Post_L	1	1
21	9	−43	25	Precuneus	Default	Cingulum_Post_R	1	1
22	5	−50	33	Precuneus	Default	Precuneus_R	1	1
23	−5	−52	17	Post-cungulate	Default	Precuneus_L	1	1
24	10	−55	17	Post-cungulate	Default	Precuneus_R	1	1
25	−6	−56	29	Precuneus	Default	Precuneus_L	1	1
26	−11	−58	17	Post-cungulate	Default	Cuneus_L	1	1
27	51	−59	34	Angular gyrus	Default	Angular_R	1	2
28	−48	−63	35	Angular gyrus	Default	Angular_L	1	1
29	11	−68	42	Precuneus	Default	Precuneus_R	2	2
30	−36	−69	40	IPS	Default	Parietal_Inf_L	1	1
31	−9	−72	41	Occipital	Default	Precuneus_L	2	1
32	45	−72	29	Occipital	Default	Occipital_Mid_R	1	2
33	−2	−75	32	Occipital	Default	Cuneus_L	1	1
34	−42	−76	26	Occipital	Default	Occipital_Mid_L	1	1
35	29	57	18	aPFC	Fronto-parietal	Frontal_Mid_R	3	3
36	−29	57	10	aPFC	Fronto-parietal	Frontal_Mid_L	3	3
37	42	48	−3	vent aPFC	Fronto-parietal	Frontal_Inf_Tri_R	2	3
38	−43	47	2	vent aPFC	Fronto-parietal	Frontal_Inf_Tri_L	2	2
39	39	42	16	vlPFC	Fronto-parietal	Frontal_Mid_R	2	3
40	40	36	29	dlPFC	Fronto-parietal	Frontal_Mid_R	2	2
41	−1	28	40	ACC	Fronto-parietal	Frontal_Sup_Medial_L	3	3
42	46	28	31	dlPFC	Fronto-parietal	Frontal_Mid_R	2	2
43	−52	28	17	vPFC	Fronto-parietal	Frontal_Inf_Tri_L	2	2
44	−44	27	33	dlPFC	Fronto-parietal	Frontal_Mid_L	2	2
45	40	17	40	dFC	Fronto-parietal	Frontal_Mid_R	1	2
46	44	8	34	dFC	Fronto-parietal	Precentral_R	2	2
47	−42	7	36	dFC	Fronto-parietal	Precentral_L	1	2
48	−41	−40	42	IPL	Fronto-parietal	Parietal_Inf_L	2	2
49	54	−44	43	IPL	Fronto-parietal	Parietal_Inf_R	2	2
50	−35	−46	48	Post-parietal	Fronto-parietal	Parietal_Inf_L	2	2
51	−48	−47	49	IPL	Fronto-parietal	Parietal_Inf_L	2	2
52	−53	−50	39	IPL	Fronto-parietal	Parietal_Inf_L	2	2
53	44	−52	47	IPL	Fronto-parietal	Parietal_Inf_R	2	2
54	−32	−58	46	IPS	Fronto-parietal	Parietal_Inf_L	2	2
55	32	−59	41	IPS	fronto-parietal	Angular_R	2	2
56	27	49	26	aPFC	Cingulo-opercular	Frontal_Mid_R	3	3
57	34	32	7	vPFC	Cingulo-opercular	Frontal_Inf_Tri_R	2	3
58	−2	30	27	ACC	Cingulo-opercular	Cingulum_Mid_L	3	3
59	51	23	8	vFC	Cingulo-opercular	Frontal_Inf_Tri_R	1	2
60	38	21	−1	Ant insula	Cingulo-opercular	Insula_R	3	3
61	9	20	34	dACC	Cingulo-opercular	Cingulum_Mid_R	3	3
62	−36	18	2	Ant insula	Cingulo-opercular	Insula_L	3	3
63	−6	17	34	Basal ganglia	Cingulo-opercular	Cingulum_Mid_L	3	3
64	0	15	45	mFC	Cingulo-opercular	Supp_Motor_Area_L	3	3
65	−46	10	14	vFC	Cingulo-opercular	Rolandic_Oper_L	2	2
66	−20	6	7	Basal ganglia	Cingulo-opercular	Putamen_L	3	3
67	14	6	7	Basal ganglia	Cingulo-opercular	Caudate_R	3	3
68	−48	6	1	vFC	Cingulo-opercular	Insula_L	3	3
69	37	−2	−3	Mid insula	Cingulo-opercular	Putamen_R	3	3
70	−12	−3	13	Thalamus	Cingulo-opercular	Caudate_L	3	3
71	−12	−12	6	Thalamus	Cingulo-opercular	Thalamus_L	3	3
72	11	−12	6	Thalamus	Cingulo-opercular	Thalamus_R	3	3
73	32	−12	2	Mid insula	Cingulo-opercular	Putamen_R	3	3
74	−30	−14	1	Mid insula	Cingulo-opercular	Putamen_L	3	3
75	11	−24	2	Basal ganglia	Cingulo-opercular	Thalamus_R	3	3
76	−30	−28	9	Post−insula	Cingulo-opercular	Heschl_L	3	3
77	51	−30	5	Temporal	Cingulo-opercular	Temporal_Sup_R	1	1
78	−4	−31	−4	Post-cungulate	Cingulo-opercular	Thalamus_L	3	3
79	54	−31	−18	Fusiform	Cingulo-opercular	Temporal_Mid_R	1	1
80	8	−40	50	Precuneus	Cingulo-opercular	Precuneus_R	1	3
81	58	−41	20	Parietal	Cingulo-opercular	Temporal_Sup_R	1	1
82	43	−43	8	Temporal	Cingulo-opercular	Temporal_Mid_R	1	1
83	−55	−44	30	Parietal	Cingulo-opercular	SupraMarginal_L	2	2
84	42	−46	21	Sup temporal	Cingulo-opercular	Angular_R	1	1
85	−41	−47	29	Angular gyrus	Cingulo-opercular	Angular_L	1	2
86	−59	−47	11	Temporal	Cingulo-opercular	Temporal_Mid_L	1	1
87	−52	−63	15	TPJ	Cingulo-opercular	Temporal_Mid_L	1	1

In the column assigned “Dosenbach and network,” ROI labels and network assignment of ROIs in Dosenbach et al. (2010) are shown. In the column assigned “AAL,” corresponding labels obtained from automated anatomical labeling (AAL) (Tzourio-Mazoyer et al., 2002) are listed. We found three communities in frequency-specific networks in the VLF and LF. In the column assigned “community,” the community assignment of each ROI is indicated by the number of assigned communities in each frequency band (VLF and LF).

### Detection of frequency specificity for functional connectivity

**Figure 1:**
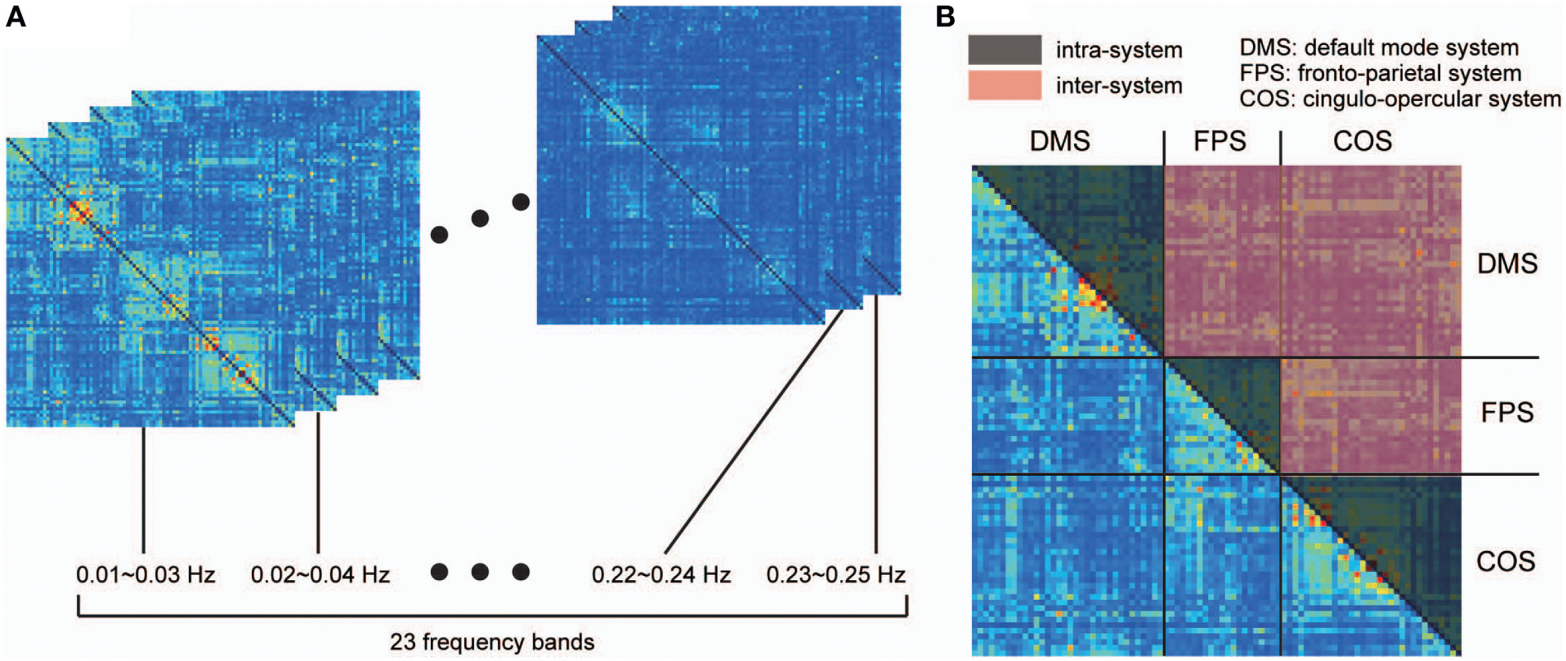
**Procedure for detecting frequency specificity of functional connectivity**. Coherence was estimated in all pairs of ROIs between 0.01 and 0.25 Hz, and averaged within narrow, 50%-overlapping frequency bands that had a band width of 0.02 Hz. As a result, we obtained coherence matrices from 23 frequency bands **(A)**. **(B)** Considering that frequency specificity is different between ROIs in the same functional system and ROIs of two distinct functional systems, coherence values were divided into two categories: coherence values within the same functional system (intra-system), and coherence values between different functional systems (inter-system). The colored parts of the matrix in **(B)** correspond to the coherence values of the intra-system (black) and inter-system (red). To investigate frequency specificity in these categories, the coherence values were further averaged within each category in each coherence matrix.

For each individual data set, we calculated the coherence between all pairs of signals extracted from the above-mentioned 87 ROIs with radii of 6 mm. Coherence measures the linear and time-invariant relationship between two signals at frequency λ and is defined as follows:

$$C_{xy}(\lambda )=\frac{{\left|P_{xy}(\lambda )\right|}^{2}}{P_{xx}(\lambda )P_{yy}(\lambda )},$$

where *C*_*xy*_(λ) refers to the coherence between signals *x* and *y*, *P*_*xy*_(λ) is the cross-spectrum of *x* and *y*, *P*_*xx*_(λ) is the power spectrum of *x*, and *P*_*yy*_(λ) is the power spectrum of *y*. For each pair of signals, we obtained coherence matrices by averaging the coherence values within 23 narrow, 50%-overlapping frequency bands, with bandwidths of 0.02 Hz (Figure 1A). Chang and Glover (2010) showed that the frequency dependency of coherence among the ROIs organizing the DMS is different from those estimated based on ROIs of two distinct functional systems (DMS and dorsal attention systems). Taking this into consideration, we further averaged these band-averaged coherence values within two categories of ROI pairs in order to identify the frequency-dependency of functional connectivity: ROIs within in the same functional system (intra-system), and ROIs belonging to different functional systems (inter-system; Dosenbach et al., 2010; Figure 1B). We then identified and conducted analyses on the frequency bands showing higher coherence values than other bands in both spectrums obtained from the averaged coherence in the two categories.

In order to confirm that the high coherence detected by the above-mentioned processes was not generated by aliasing of the physiological confounds contained in the hemodynamic signals, and could not be attributed to measurement modality (e.g., fMRI), we investigated coherence spectrums with NIRS signals. By projecting vitamin tablets onto cortical surfaces from structural MR images, we identified ROIs where NIRS signals were obtained. Two ROIs were identified within brain regions constituting the fronto-parietal system, and one ROI was located within the brain region forming the default mode system. In order to compare the results with those obtained using fMRI, we included in our analyses only the three NIRS channels located within brain regions included in the abovementioned resting-state functional connectivity systems. We then calculated the coherence between two ROIs in the fronto-parietal system and among ROIs between the fronto-parietal and default mode systems as counterparts of coherence for intra- and inter-systems.

### Graph metrics for segregation and integration

Network structures can be indices of the degree of information segregation and integration. For example, communities are sets of nodes in a network with dense interconnections. Their existence in a network enhances both communications within individual communities and independences between different communities—that is, segregation. Metrics that quantify how clearly communities in a network segregate can be used to investigate the degree of information segregation. By contrast, network structures such as hubs, which promote efficient communication among different nodes, can be indices of information integration. In another words, we can quantify the degree of information integration using graph metrics that measure how efficiently the nodes in a network communicate with each other. In the present study, we selected modularity as a measure of information segregation, and global efficiency as a measure of information integration.

#### Frequency-specific network construction in individual data sets

To define frequency-specific networks, we obtained adjacency matrices *A* by applying the sparsity thresholds *S*, ranging from 0.05 to 0.25 in 0.05 increments, to the coherence matrices corresponding to the frequency bands showing higher coherence values than others (see above). Coherence matrices were calculated by averaging the coherence values within the frequency bands, which are a part of the matrices shown in Figure 1A. Sparsity *S* was defined as the number of edges in a graph, divided by the maximum possible number of edges, and was used to measure the threshold (Latora and Marchiori, 2001; Achard and Bullmore, 2007):

$$S=\frac{1}{n(n-1)}\;\sum_{i\in N}d_{i},$$

where *n* is the number of nodes in a graph *N*, *i* is a node in graph *N*, and *d*_*i*_ is the number of edges connected to the node *i*. Unlike a threshold using the value quantifying the strength of functional connectivity, *S* can control the number of edges in the network between different conditions. Because many network metrics are affected by the number of edges in a graph, using *S* allows us to attribute the different results of graph measures to differences in patterns of network connections. When one *S* is selected, the corresponding thresholding value of the strength of functional connectivity is determined. Therefore, the range of *S* should be determined so that the corresponding thresholding value of the measure of functional connectivity is significantly higher than 0. To choose the lower bound of *S*, we calculated the null distributions of 10,000 coherence values by repeating calculations of shuffled signals obtained by a bootstraping method for each ROI pair. By ensuring the statistical significance (*p* < 0.05) of the coherence values corresponding to the sparsity thresholds for all participant data sets, we selected the lower bound of the sparsity thresholds as *S* = 0.25.

#### Modularity

Modularity *Q* (Newman, 2004, 2006) indicates the extent to which the network can be subdivided into non-overlapping communities. For a set of non-overlapping communities *M*, *Q* is defined as:

$$Q=\sum_{u\in M}\;\left[e_{uu}-{\left(\sum_{v\in M}e_{uv}\right)}^{2}\right],$$

where by *u* and *v* are communities, and *e*_*uv*_ is the fraction of all links that connect nodes in *u* with nodes in *v*.

#### Global efficiency

Global efficiency *E* (Latora and Marchiori, 2001) is an indicator of the global efficiency of parallel information transfer in the network and is defined as follows:

$$E=\frac{1}{n}\;\sum_{i\;\in \;N}\;\frac{\sum_{j\;\in \;N,\;j\;\neq \;i}d_{ij}^{-1}}{n-1}.$$

In this case, *n* is the number of nodes in the network, *N* is the set of all nodes, and *d_*ij*_* is the shortest path length between node *i* and node *j*.

All of these metrics were computed for each sparsity threshold in each individual data set using the Brain Connectivity Toolbox (Rubinov and Sporns, 2010).

#### Statistical comparisons

For each threshold level, we conducted a two-tailed *t*-test with subjects (random effects analysis) against the null hypothesis, defined as no significant group-level difference between graph metrics calculated in two frequency bands that showed higher coherence values than other bands. Because we used 5 different sparsity thresholds and 2 graph theoretical metrics, correction was conducted with considering 10 multiple comparisons. The false discovery rate (FDR) method was used to correct for multiple comparisons, and significant differences were detected at *p* < 0.05 after FDR correction (Benjamini and Yekutieli, 2001).

### Identification of frequency-specific network structures for segregation and integration

Graph metrics can reveal network attributes for information segregation and integration; however, it is not clear which network structures realize these informational properties. Brain networks possess characteristic structures that play key roles in segregation and integration; yet it remains unclear whether these structures can be consistently found in frequency-specific networks. To tackle this issue, we estimated group-level network structures. For each group-level frequency-specific network, we identified communities as structures for information segregation, and hubs and rich-clubs (higher-order structures of hubs) as structures for information integration.

#### Group-level network construction

Network connection patterns have inter-individual variability. In order to investigate consistencies in structure at the group level, we constructed a network-level adjacency matrix *A*^*g*^ from the individual-level adjacency matrices *A* of all individual data sets. *A* is a binary matrix, defined as *A*_*ij*_ = 1 when there is functional connectivity between node *i* and *j*, otherwise it is defined as *A*_*ij*_ = 0. Adjacency matrix *A* was calculated for each frequency band in each participant's dataset. We generated a matrix representing the consistency of functional connectivity across all participants by averaging *A* in each frequency band. We refer to this here as the consistent edge matrix *Ce* (0 ≦ *Ce*_*ij*_ ≦ 1). Then, by applying sparsity thresholds on *Ce*, we obtained *A*^*g*^ in frequency bands where the coherence showed higher values than other frequency bands in both spectrums obtained from the averaged coherence in the two categories. Although the same sparsity thresholds *S* between 0.05 and 0.25 for the 0.05 increments were used in producing *A* and *A*^*g*^, we finally selected *Ss* generating connected *A^*g*^*s, which are graphs in which there is at least one direct or indirect pathway among all nodes, for all frequency bands showing higher coherence values than others.

#### Community detection

We then examined group-level community structures in group-level networks obtained in different frequency bands. As community structures have between-participant variability, group-level community structures were not identified with the consistent edge matrix *Ce*, but rather were detected with the consistent assignment matrix *Ca* (van den Heuvel et al., 2008a; Fornito et al., 2012). This matrix was constructed as follows. First, community detection was conducted on the adjacency matrix *A* of each participant. Individual-level consistency of community assignment was expressed in a matrix *ICa* in which the element *ICa*_*ij*_ is 1 if the ROIs *i* and *j* are assigned in the same community. Then, *ICa*s were averaged across participants to produce *Ca*s in which the element *Ca*_*ij*_ represents the incidence of two ROIs being assigned to an identical community within the group (0 ≦ *Ca_*ij*_* ≦ 1). Finally, by applying the community detection algorithm on *Ca*s, we estimated group-level community structures in all frequency bands with higher coherence values than others. Several algorithms have been developed to identify optimal communities in a network. We compared the effectiveness of three community detection algorithms (Newman, 2006; Blondel et al., 2008; Sun et al., 2009) implemented in the Brain Connectivity Toolbox to estimate community structures. The Louvein method algorithm (MATLAB function in Brain Connectivity Toolbox, modularity_louvain_und.m) was selected for detecting communities as it returned the highest modularity.

Once communities were detected in each frequency-specific network, we assessed the similarity of the brain regions, formed by using normalized mutual information *NMI*, as proposed by Kuncheva and Hadjitodorov (2004), to quantify the similarity of different community assignments on the same node set (Meunier et al., 2009). *NMI* can quantify the accuracy with which one assignment of a given node set predicts the other, and it is defined as:

$$NMI=\frac{-2\;\sum_{i\;=1}^{c_{1}}\;\sum_{j\;=1}^{c_{1}}N_{ij}\;\log(\frac{N_{ij}N}{N_{i}N_{j}})}{\sum_{i\;=\;1}^{c_{1}}N_{i}\;\log\;(\frac{N_{i}}{N})+\sum_{j\;=\;1}^{c_{2}}N_{j}\log\;(\frac{N_{j}}{N})}.$$

where *C*_1_ and *C*_2_ are the number of detected communities for each assignment, *N* is the number of nodes in the network, *N*_*i*_ and N_*j*_ are the numbers of nodes assigned in *i*-th and *j*-th communities, and *N*_*ij*_ is the number of nodes commonly assigned in both *N_*i*_* and *N_*j*_* in different partitions. The same assignments give *NMI* = 1, while *NMI* is 0 among totally independent assignments.

Statistical significance for the similarity between assignments of VLF and of LF was tested by generating 10,000 pairs of community assignments in randomized networks of *A*^*g*^s. This produced a null distribution consisting of the 10,000 *NMI*s. In addition, we calculated the *NMI* between the frequency-specific networks and community assignment reported in Dosenbach et al. (2010). The adjacency matrix in which the community assignment was calculated in Dosenbach et al. (2010) was not reported; therefore, we obtained the null distribution of the community assignment by generating 10,000 random networks in addition to 10,000 randomized *A*^*g*^s, in order to test the statistical significance of the *NMI*s.

#### Hub detection

We detected hub regions in all group-level networks obtained in different frequency bands, in order to assess whether topological differences were reflected in different hub alignments between these networks. In order to identify hub regions, we measured two graph theoretical metrics for each node: the nodal degree and the eigenvector centrality (Lohmann et al., 2010). The nodal degree was calculated in group-level adjacency matrices *A*^*g*^s, whereas the eigenvector centrality was computed in consistent edge matrices *Ce*s without applying a threshold. In the current study, we defined hubs as nodes in which both the nodal degree and the eigenvector centrality were at least one standard deviation above the network mean. The nodal degree and eigenvector centrality measure different aspects of nodes in the network; the nodal degree simply counts the number of edges connecting a node, whereas the eigenvector centrality estimates how a node can affect others connecting with it. As we aimed to identify hub regions with highly connected and highly central nodes in a network, we used both criteria to define them.

#### Rich-club detection

A study of human anatomical connectivity has shown that structural brain hubs are not independent of each other, but rather form a rich club. This is characterized by a tendency for high-degree nodes to be more densely anatomically connected among themselves than nodes of a lower degree (van den Heuvel and Sporns, 2011). The appearance of a rich club in human anatomical networks suggests that these regions, identified as structural brain hubs, perform some collaborative functions like information integration. This raises a question of whether hubs identified in FCNs also organize the rich-club.

By denoting the number of nodes with a higher degree than *k* as *N*_*k*_, and designating the edges within the sub-network that consist of these nodes as *E*_*k*_, *k*-density Φ(*k*) is defined as follows:

$$\varphi (k)=\frac{2E_{k}}{N_{k}(N_{k}-1)}.$$

The denominator represents the maximal number of edges within the sub-network. Several graphs including a random network, in which nodes are interconnected by chance, show that Φ(*k*) grows with *k*. Therefore, if there is a tendency for hubs to be more inter-connected than nodes of a lower degree, Φ(*k*) increases with *k* at a higher rate than those expected out of random networks (Colizza et al., 2006; McAuley et al., 2007)—that is, Φ(*k*) is informative when this coefficient is normalized by the expected one. Therefore, we identified a range of *k*-values expressing this characteristic as follows, and subsequently refer to such phenomena as a “rich-club regime” (van den Heuvel and Sporns, 2011). First, we calculated Φ(*k*) for all *A*^*g*^s. Then, we constructed 1000 randomized networks for each of the *A*^*g*^s using the Brain Connectivity Toolbox, and computed 1000 coefficients in these networks Φ_*randomized*_(*k*). We defined the range of *k* where Φ(*k*) was significantly higher than the values calculated in the randomized networks. To evaluate statistical significance, we compared Φ(*k*) with the distribution consisting of 1000 Φ_*randomized*_(*k*)s and identified the range where Φ(*k*)s were consistently included within the upper 1% of the distribution. When we detected more than two ranges satisfying this condition, we defined a rich-club regime as the highest range. Finally, normalized rich-club coefficients Φ_*normalized*_(*k*) were calculated by dividing Φ(*k*) with Φ_*meanrand*_(*k*), which represents the mean of 1000 Φ_*randomized*_(*k*)s.

#### Characterizing the functional role of hubs

The functional role of a hub in a network can be determined via the relationship with communities. For example, hubs with connections that are mostly within a single community (intra-community connections) facilitate integration within that community, whereas those with multiple connections to different communities (inter-community connections) promote integration among communities. The participation coefficient *P* is a graph theoretical metric that expresses the distribution of intra- vs. inter-community connections (Guimera and Amaral, 2005). The *P*-value of an individual node, *P*_*i*_, is defined as follows:

$$P_{i}=1-\sum_{s\;=\;1}^{N_{M}}{\left(\frac{\kappa_{is}}{k_{i}}\right)}^{2},$$

where N_*M*_ is the number of identified communities in a network, *k*_*i*_ is the degree of the node, and κ_*is*_ is the number of edges linking the node and other nodes within a community expressed by the subscript *s*. Guimera and Amaral (2005) have shown that, by using this metric, hubs can be naturally divided into three different roles: provincial hubs (*P* ≤ 0.30), connector hubs (0.30 < *P* ≤ 0.75), and kinless hubs (*P* > 0.75). The application of this metric in brain networks has successfully characterized the functional roles of hubs in cats, macaques, and humans (Sporns et al., 2007; Hagmann et al., 2008; Meunier et al., 2009; Power et al., 2013). In the current study, we used this metric to characterize the functional role of detected hubs in frequency-specific FCNs.

## Results

### Frequency-dependency of functional connectivity

**Figure 2:**
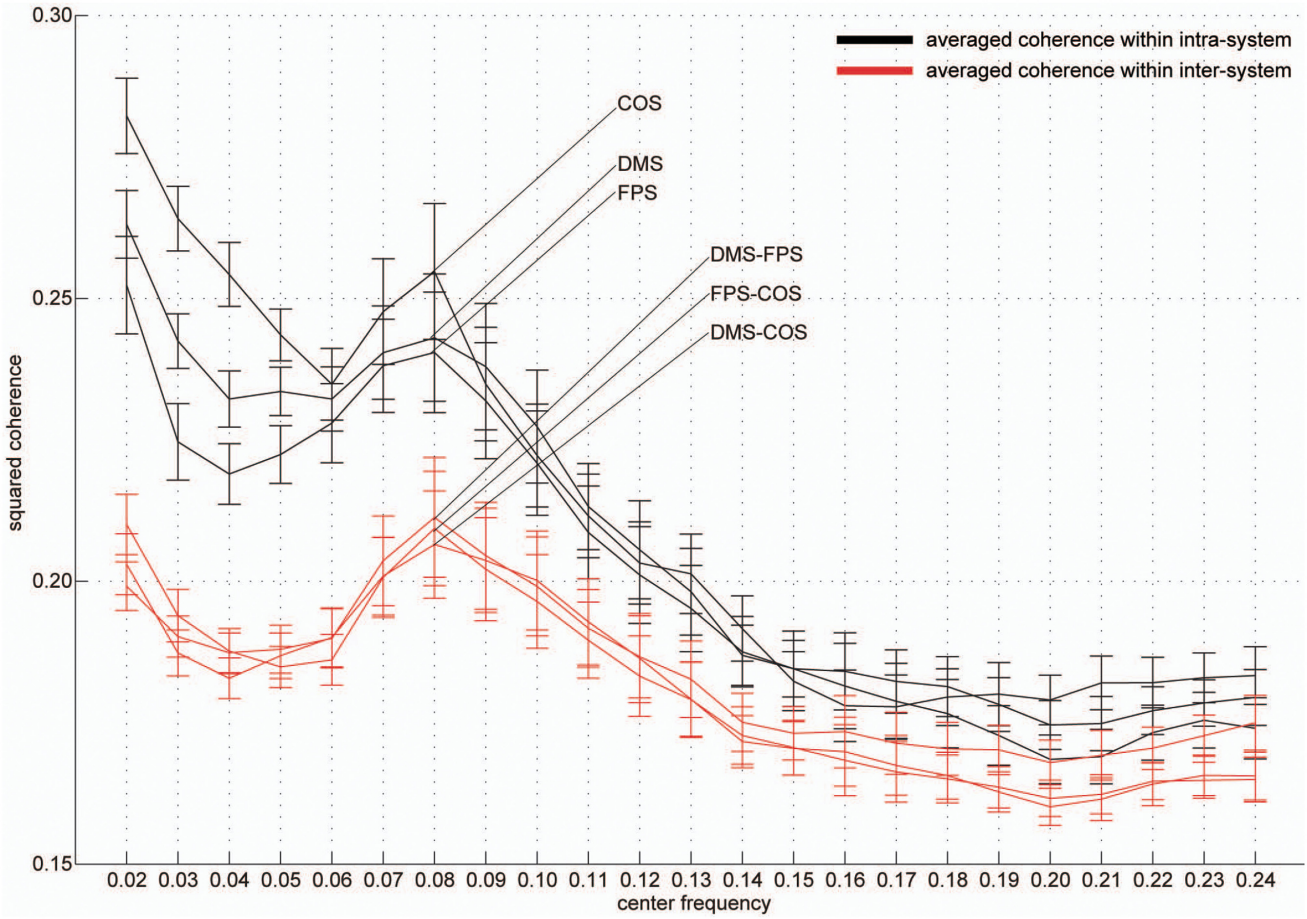
**Frequency-specificity of functional connectivity**. Averaged coherence values in two categories are shown. Black curves represent coherence values averaged within three functional systems and red curves indicate values calculated in the inter-system groups (Figure 1). Error bars show the standard errors. The x-axis represents the center frequencies of the frequency bands, where coherence values were averaged. For all curves, coherence within 0.01–0.03 Hz (very low frequency, [VLF]) and 0.07–0.09 Hz (low frequency, [LF]) were higher than those of other frequency bands.

**Figure 3:**
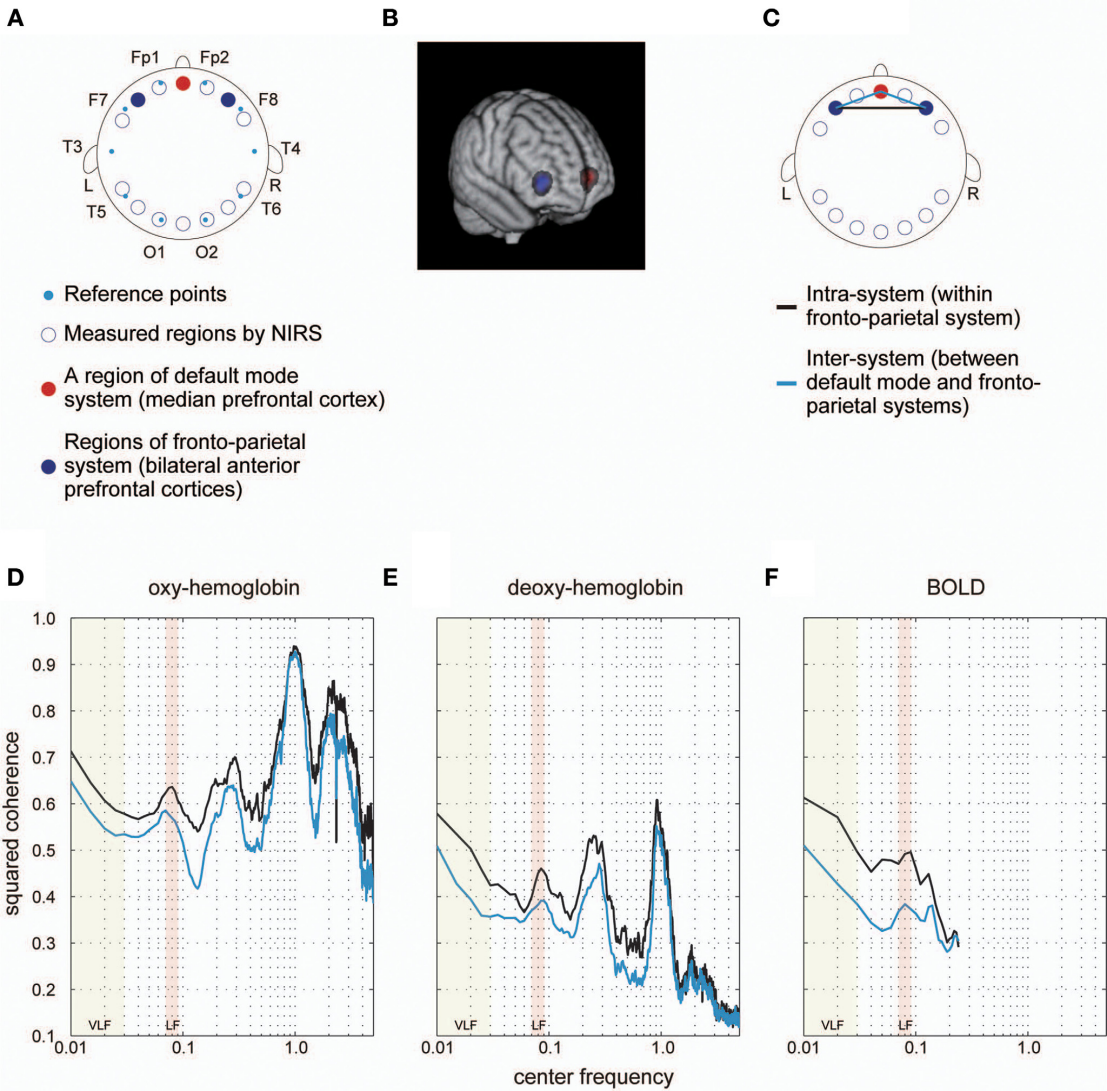
**Coherence spectrum estimated using simultaneously obtained NIRS data. (A)** We obtained NIRS signals at 14 cortical regions indicated as blue rings. Cyan dots represent standard reference points used in locating channels of electroencephalography on the scalp. In a previous study, we identified 14 cortical regions, where NIRS signals were obtained, in MNI space to determine ROIs corresponding to each NIRS measurement region for each individual. For detailed methods for the identification and MNI coordinates, please refer to Sasai et al. (2012). As a result, we found one cortical region (medial prefrontal cortex [mPFC]) included in the default mode system (red filled circle) and two bilateral cortical regions (left and right anterior prefrontal cortices [laPFC and raPFC]) contained in the fronto-parietal system (blue filled circles). **(B)** Voxels corresponding to measured regions by NIRS are shown. Colors are the same as those defined in **(A)**. **(C)** We calculated the coherence between laPFC and raPFC to investigate the intra-system coherence spectrum (fronto-parietal system), and also estimated the coherence between mPFC and laPFC, and between mPFC and raPFC, to examine the inter-system coherence spectrum (default-mode and fronto-parietal systems). A black line indicates an intra-system pair of ROIs, whereas cyan lines represent inter-system pairs. **(D,E)** Coherence spectrums of two NIRS signals (oxygenated [oxy-] hemoglobin and deoxygenated [deoxy-] hemoglobin) with two clear peaks corresponding to typical frequency bands of respiratory fluctuation around 0.3 Hz and cardiac pulsations around 1 Hz. High coherences in VLF and LF could still be observed in the spectrum, supporting the idea that higher coherences in these bands are not due to aliasing. **(F)** Coherence spectrum obtained using fMRI signals extracted from ROIs corresponding to NIRS measurement regions (as shown in **B**). We confirmed the high coherence values in VLF and LF in this spectrum, supporting the notion that characteristics of the coherence spectrum cannot be attributed to differences in ROI locations between our current and previous studies.

Group-averaged coherence values were calculated and averaged within the following two categories of ROI pairs: ROIs within the same functional system (intra-system) and ROIs belonging to different functional systems (inter-system; Dosenbach et al., 2010; Figure 1B). The highest value of the averaged coherence in the intra-system was observed in the lowest frequency band; hereafter we refer to this frequency band (0.01–0.03 Hz) as VLF (very low frequency) represented by its center frequency (0.02 Hz) in Figure 2. There was one other frequency band in which the coherence values were higher than others; we call this frequency band (0.07–0.09 Hz) as the LF (low frequency band) indicated as 0.08 Hz in Figure 2. In both frequency bands, we found that the averaged coherence value obtained in the inter-system was also higher than other frequency bands. The result did not depend on the width of frequency bands where coherence values were averaged (Figure S1). In order to ensure that the observed frequency characteristics were reproducible in data sets obtained at other institutions, we estimated the above-mentioned coherence spectrum in a public resting state fMRI data set with 96 participants from the 1000 Functional Connectome Project (http://fcon_1000.projects.nitrc.org/index.html). Results confirmed that coherence values in the VLF and LF were larger than those in other frequency bands (Figure S2). Furthermore, to confirm that this frequency specificity was not due to aliasing of physiological noise contained in higher frequency regions, we investigated the coherence spectrum of a simultaneously obtained NIRS data set. NIRS signals were measured with a sufficiently higher sampling rate (10 Hz) to characterize hemoglobin signals including respiratory and cardiac pulsations, which were observed as separate peaks in the power spectrum. We confirmed that although there were peaks corresponding to typical respiratory and cardiac pulsations around 0.3 and 1 Hz in the coherence spectrum, VLF and LF were still signature frequency bands where the coherence values were higher than other frequency bands within the 0.01–0.10 Hz band (Figure 3). Collectively, these results demonstrate that there are two frequency components that strongly contribute to resting state functional connectivity within the frequency band (0.01–0.10 Hz), where functional connectivity has been estimated in many studies. Thus, we focused and conducted analyses on these two frequency bands.

### Graph metrics for segregation and integration

**Figure 4:**
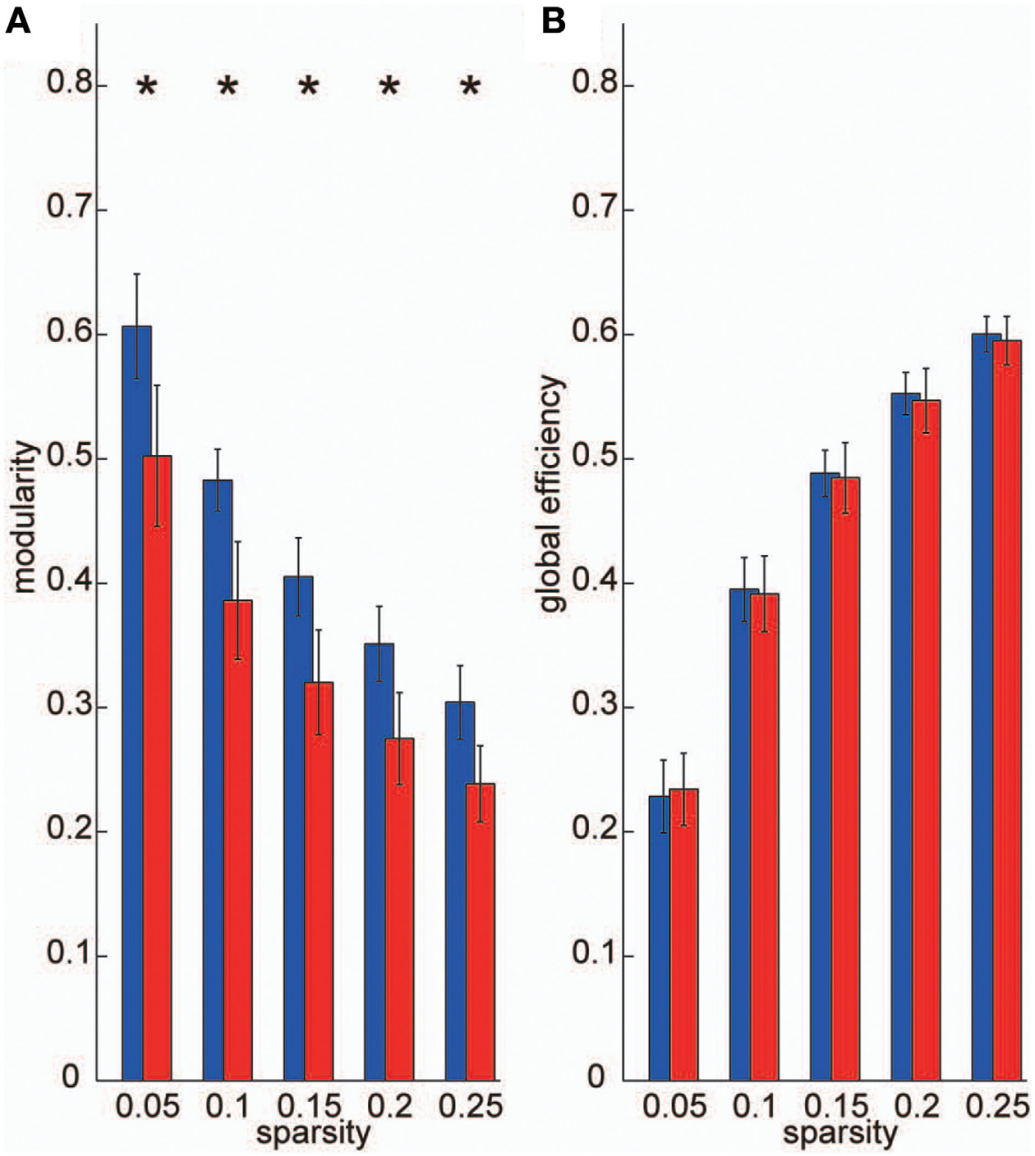
**Graph metrics**. We calculated the following two graph metrics in two frequency-specific networks (VLF and LF) estimated in each individual data set: **(A)** modularity, and **(B)** global efficiency. Blue bars represent the mean of each graph metric obtained, which was computed in networks estimated in the VLF. Red bars indicate the mean graph metric in the LF. In the current study, we selected sparsity of the brain networks (number of existing edges over the maximum possible number of edges) as threshold measurements. Because different threshold values might affect these graph metrics, we examined the between-group differences in these parameters over a wide range of threshold levels (0.05–0.25). Asterisks indicate statistically significant differences between the metrics obtained in the VLFN and the LFN, tested by two-sampled *t*-tests (*p* < 0.05, false discovery rate-corrected).

To construct frequency-specific networks identified in the VLF and LF bands for each individual data set (*I*_VLF_ and *I*_LF_), we applied five sparsity thresholds to two band-averaged coherence matrices, and calculated graph theoretical metrics using the adjacency matrices *A*. We then conducted two-tailed *t*-tests against the null-hypothesis that there were no group-level differences between *I*_VLF_ and *I*_LF_ in the calculated graph metrics. For all sparsity levels, the modularity, which is a graph metric of segregation, obtained in the *I*_VLF_ was significantly higher than in the *I*_LF_ (*p* < 0.05, corrected; Figure 4A); this demonstrated that the *I*_VLF_ had a significantly higher capacity for information segregation than the *I*_LF_. By contrast, no significant differences were found in global efficiency (a measure of information integration; Figure 4B).

### Frequency-specific network structures for segregation

We thresholded the consistent edge matrix *Ce* by applying the sparsity thresholds *S*, ranging from 0.05 to 0.25 in 0.05 increments, to obtain group-level frequency-specific networks, *A*^*g*^, in both the VLF and LF ranges (*A*^*g*^_VLF_ and *A*^*g*^_LF_). It was only when we applied *S* = 0.25 as a threshold that both *A*^*g*^_VLF_ and *A*^*g*^_LF_ became a connected graph. Therefore, we conducted the following analyses using this sparsity threshold.

**Figure 5:**
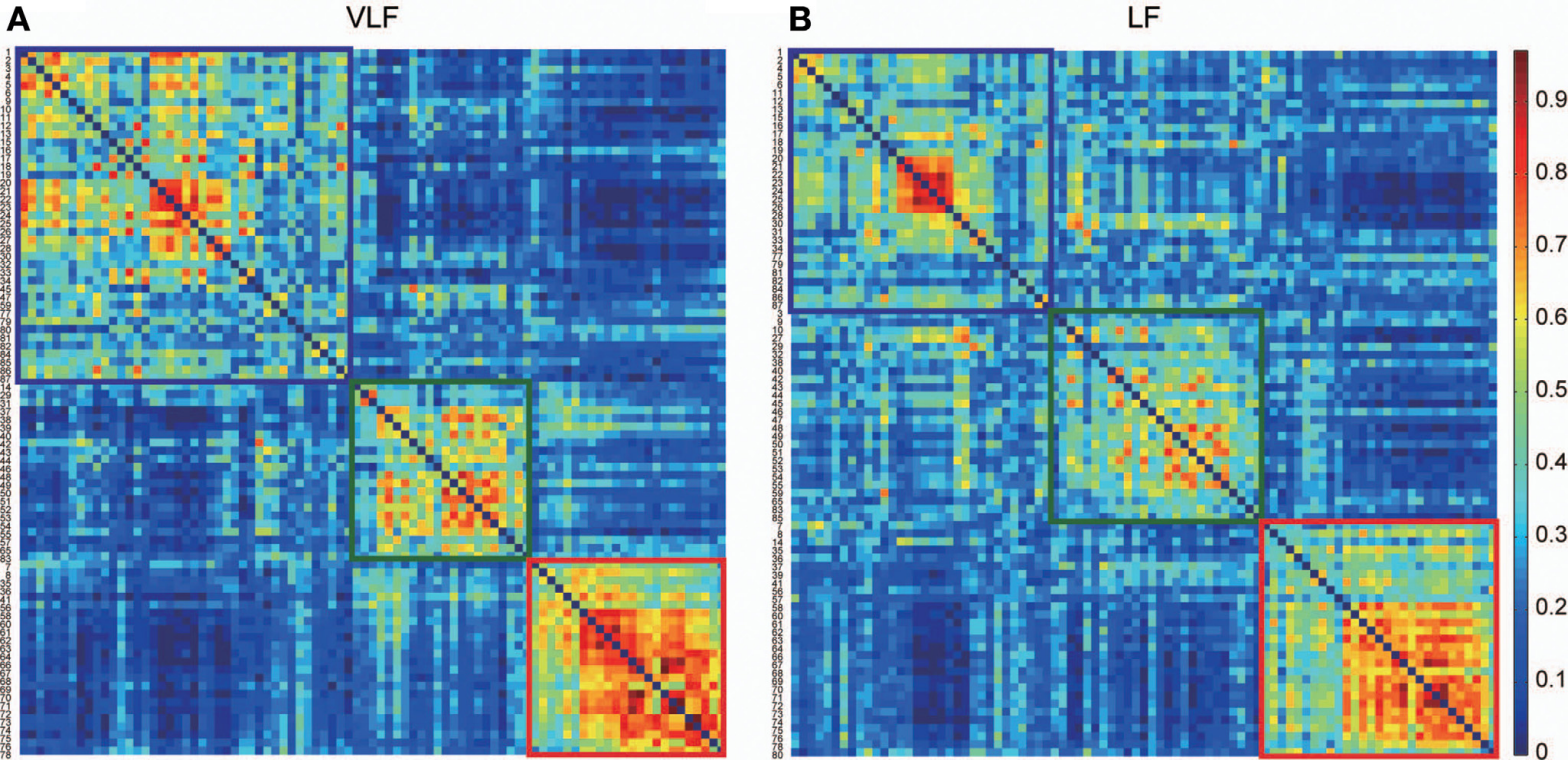
**Consistency of community detection between VLF and LF. (A,B)** Show consistent assignment matrices *Ca* obtained in the VLF **(A)** and LF **(B)**. To emphasize the modular structures, both *Ca* were reordered by putting the ROIs in the same module next to each other. Detecting communities in both matrices revealed three in the VLF and LF, indicated by squares. The color of each square corresponds to the assigned community detected in each frequency band: blue denotes community 1, green community 2, and red community 3.

By detecting consistent community structures across participants for both networks (Figures 5A,B), we found three communities in both *A*^*g*^_VLF_ and *A*^*g*^_LF_. The correspondence between each region and the assigned community is shown in Table 1. We calculated *NMI* to quantify the similarity of community assignments and found that detected communities in *A*^*g*^_VLF_ and *A*^*g*^_LF_ were significantly similar to each other (*NMI* = 0.59; *p* < 0.0001). Comparing the *NMI* between the community assignments identified in the frequency-specific networks and those reported by Dosenbach et al. (2010) revealed that assignments in both *A^*g*^*_VLF_ and *A^*g*^*_LF_ showed significant similarity with the reported assignment (0.33 for *A^*g*^*_VLF_ [*p* < 0.0001]; 0.32 for *A^*g*^*_LF_ [*p* < 0.0001]). This supports that we could consistently find three communities analogous to DMS, FPS, and COS in both *A^*g*^*_VLF_ and *A^*g*^*_LF_. (community assigned Nos. 1, 2 and 3 in Table 1). These findings suggest that, although these communities were more strongly segregated in *A^*g*^*_VLF_ than *A^*g*^*_LF_, both consisted of three communities corresponding to functional systems.

### Frequency-specific network structures for integration

**Figure 6:**
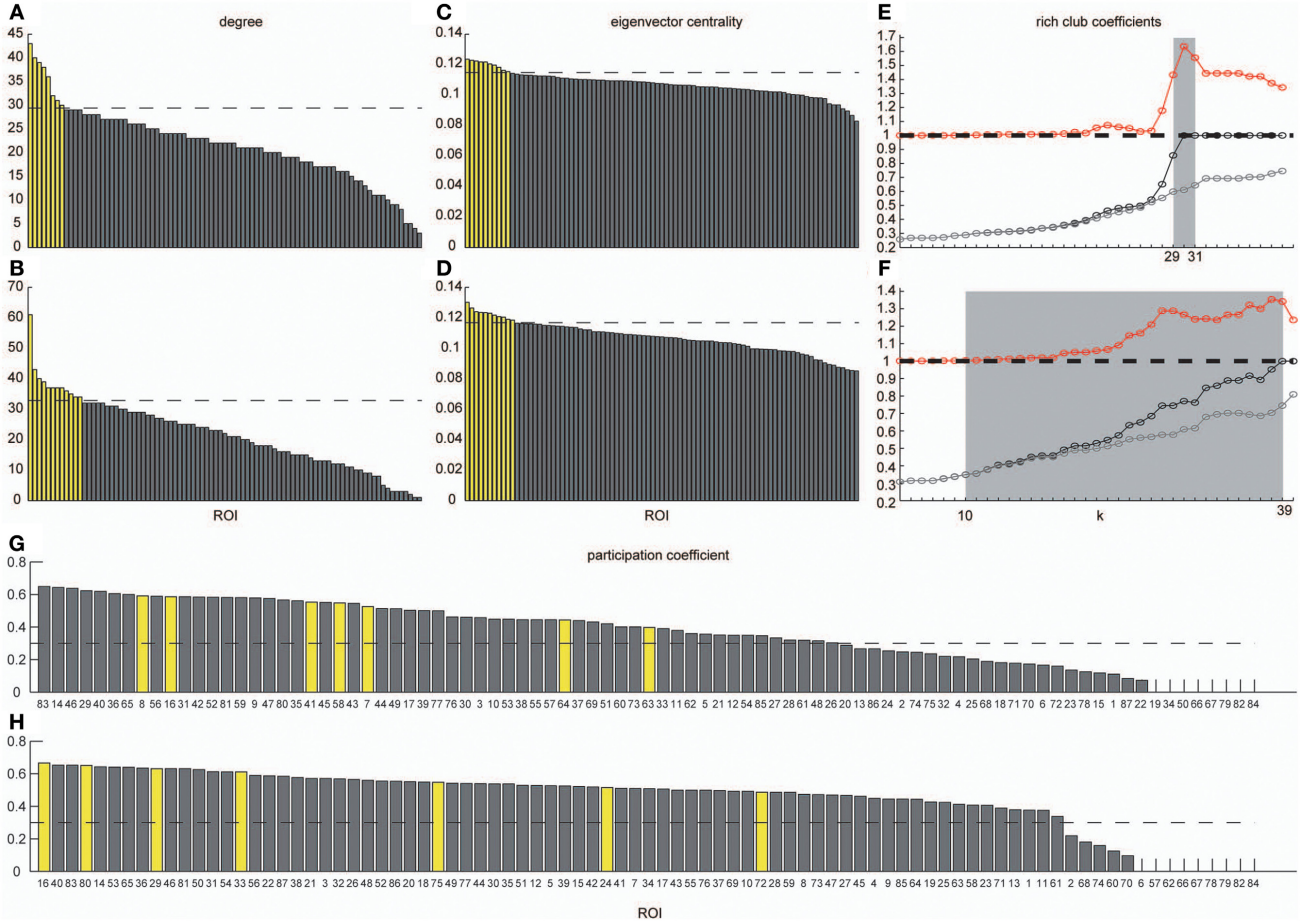
**Degrees, eigenvector centrality, rich-club coefficients and participation coefficients**. Degrees and eigenvector centralities of all nodes were calculated in the group-level network estimated in the VLF and LF. **(A)** Shows the distribution of degree estimated in the VLF, and **(B)** shows the distribution of degree estimated in the LF. **(C)** Corresponds to the distribution of eigenvector centrality estimated in the VLF, and **(D)** corresponds to the distribution of eigenvector centrality estimated in the LF. Dashed lines express the mean plus one standard deviation. Yellow bars represent nodal metrics above the threshold (the mean plus one standard deviation), and gray bars indicate those below the threshold. We also calculated rich-club coefficients in group-level frequency-specific networks (see “Rich-club detection”). Black curves correspond to Φ(*k*), gray curves correspond to Φ_*meanrand*_(*k*), and red curves correspond to Φ_*normalized*_(*k*). In both **(E,F)**, there is a tendency for Φ(*k*) to increase with *k* at a higher rate than Φ_*meanrand*_(*k*). Ranges of *k*, where Φ(*k*) became significantly higher than *Φ_*meanrand*_*(*k*), are highlighted by a gray background. **(G,H)** Show the participation coefficients of all ROIs in the VLF and LF, respectively. Yellow bars represent the coefficients of hubs in each frequency band. Broken lines indicate 0.3, which is the boundary between provincial and connector hubs (see Materials and Methods).

In order to identify hub regions in frequency-specific networks, we calculated nodal degrees and eigenvectors for *A*^*g*^_VLF_ and *A*^*g*^_LF_ (Figure 6). In both metrics, we identified high degree and high centrality nodes with metrics greater than the network mean plus one standard deviation (yellow bars in Figures 6A–D). Hub regions were then defined as ROIs detected as both high degree and high centrality nodes (Table 2). While seven ROIs were identified as hubs for both *A*^*g*^_VLF_ and *A*^*g*^_LF_, all hubs except the one for the left dorsal anterior precuneus cortex (ldaPrCC, [AAL: Cingulum_Mid_L]) were different between *A*^*g*^_VLF_ and *A^*g*^*_LF_. Frequency-specific hubs in *A*^*g*^_VLF_ were detected in the left superior medial frontal cortex, left supplementary motor area, left middle, and right anterior cingulate cortices, all of which were regions assigned in one community (No. 3 in Table 2). This consisted of similar regions to the COS. Frequency-specific hubs in *A*^*g*^_LF_ were detected in the left cuneus, right precuneus, and right thalamus. Contrary to *A*^*g*^_VLF_, the frequency-specific hubs in *A*^*g*^_LF_ were distributed over all communities.

**Table 2:** **List of hubs identified in 2 frequency-specific networks**.

**VLF**					**LF**				
**No**.	**k**	**Eigenvector**	**AAL**	**Assigned community**	**No**.	**k**	**Eigenvector**	**AAL**	**Assigned community**
**41**	1	6	Frontal_Sup_Medial_L	3	**16**	1	1	Cingulum_Mid_L	1
**16**	2	4	Cingulum_Mid_L	1	**80**	2	4	Precuneus_R	3
**7**	3	10	Cingulum_Ant_R	3	**33**	3	3	Cuneus_L	1
**58**	4	1	Cingulum_Mid_L	3	**72**	4	4	Thalamus_R	3
**8**	5	9	Cingulum_Ant_R	3	**24**	5	2	Precuneus_R	1
**64**	6	4	Supp_Motor_Area_L	3	**75**	5	7	Thalamus_R	3
**63**	7	2	Cingulum_Mid_L	3	**29**	5	9	Precuneus_R	2
45	8		Frontal_Mid_R		69	5		Putamen_R	
24		3	Precuneus_R		22	9		Precuneus_R	
61		7	Cingulum_Mid_R		36	10		Frontal_Mid_L	
23		8	Precuneus_L		5	11		Cingulum_Ant_L	
					14	12		Cingulum_Mid_R	
					71		6	Thalamus_L	
					26		8	Cuneus_L	
					63		10	Cingulum_Mid_L	
					23		11	Precuneus_L	

The hubs identified in each network are shown. To identify hub regions, we calculated the group means of the coherence values and the estimated group-averaged VLFN and LFN. We selected a sparsity of 0.25 as the threshold value, because all networks become connected in graphs only using this value. Then, a hub was defined as a node satisfying the following two conditions: first, the nodal degree was higher than the mean plus the standard deviation of the degree distribution; and second, the eigenvector centrality that was measured in a weighted non-thresholded network was higher than the mean plus the standard deviation of the distribution of the centrality. In the column assigned “No,” bold type-face shows identified hubs, and normal type-face represents non-hub nodes that have a larger nodal degree or eigenvector centrality than the thresholds. The numbers in column k and the eigenvector indicate the ranks of the nodes for the above two conditions, respectively. Anatomical labels obtained from AAL are listed in column AAL. The numbers in the column assigned community indicate those of detected communities where hubs were assigned in each frequency-specific network.

Figures 6E,F show the rich-club coefficient curves obtained in both *A^*g*^*_VLF_ and *A^*g*^*_LF_. We found a range of *k*-values showing significantly higher rich-club coefficients than those calculated in randomized topologies for both *A^*g*^*_VLF_ and *A^*g*^*_LF._ The rich-club regime in *A^*g*^*_VLF_ was 29 ≦ *k* ≦ 31 vs. 10 ≦ *k* ≦ 39 in *A^*g*^*_LF_. In the rich-club regime observed in each frequency band, we found a value of k at which the rich-club organization in each frequency-specific network was formed by a detected hub region (*k* = 30 for *A^*g*^*_VLF_ and *k* = 36 for *A^*g*^*_LF_). This demonstrated that significantly dense interconnections exist among hubs in each frequency-specific network.

The functional roles of the identified hubs were estimated by calculating the participation coefficient *P* in both *A^*g*^*_VLF_ and *A^*g*^*_LF_ (Figures 6G,H). As *P* cannot exceed 0.67 for networks consisting of three communities, we cannot observe kinless hubs (hubs with *P* > 0.75; see Materials and Methods and Guimera and Amaral, 2005) in both frequency-specific networks. We found that all detected hubs in both *A^*g*^*_VLF_ and *A^*g*^*_LF_ were classified in connector hubs (0.3 ≤ *P* < 0.75), indicating that hubs have a role in integration among the three detected communities in both *A^*g*^*_VLF_ and *A^*g*^*_LF_.

**Figure 7:**
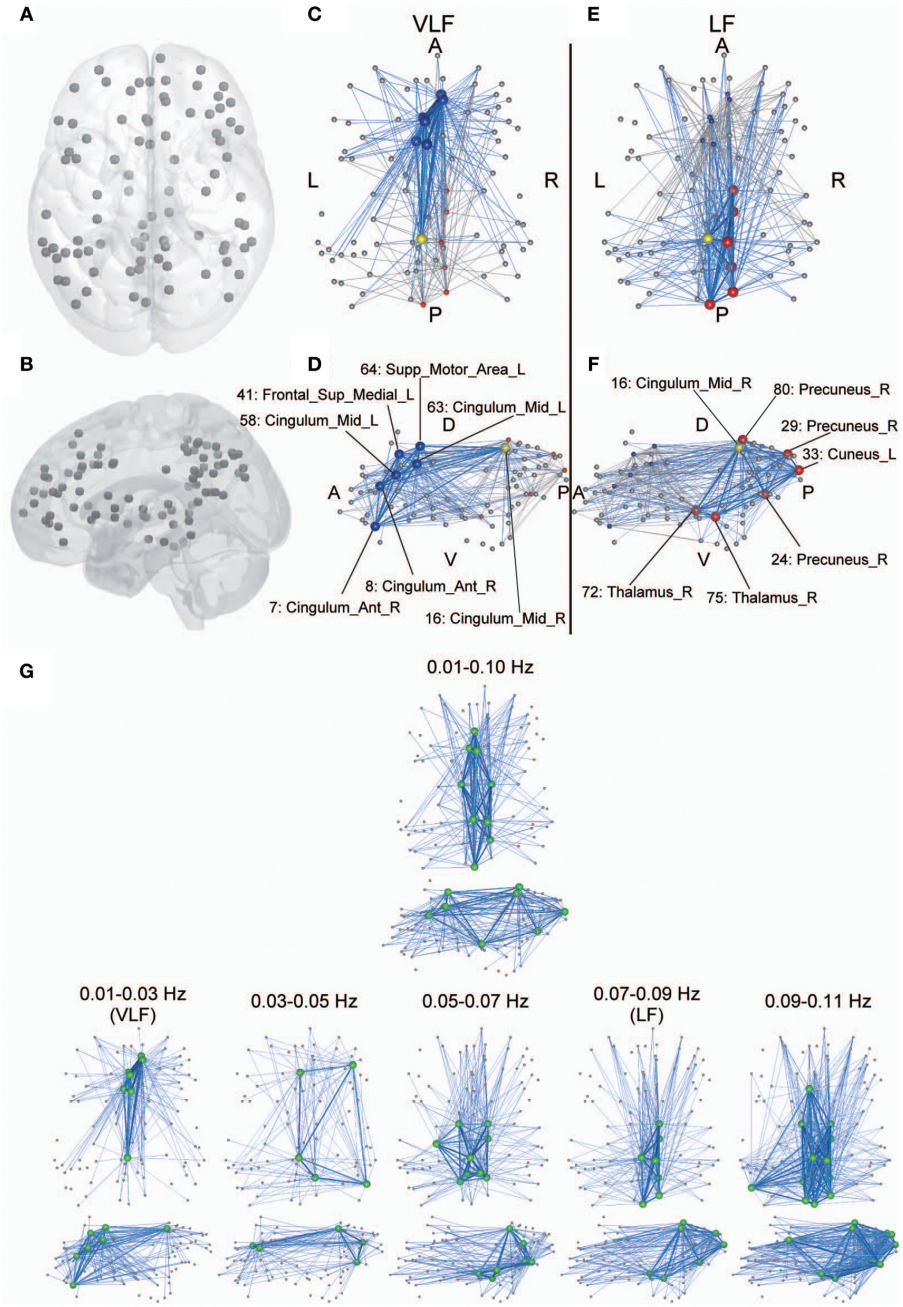
**Anatomical perspective of hub regions. (A,B)** Eighty-seven ROIs used in the current study are displayed on a surface rendering of the brain using MATcro software distributed by http://www.mccauslandcenter.sc.edu/CRNL/tools/surface-rendering-with-matlab. Hub regions in frequency-specific networks were the seven highest degree nodes in each frequency band **(C–F**). The yellow node is a hub that is consistently identified in both the VLF **(C,D)** and the LF **(E,F)**. The blue nodes are hubs identified only in the VLF, and the red nodes are those identified only in the LF. Hubs are represented by large spheres. Blue lines indicate functional connectivity with hubs within the frequency band, and gray lines represent functional connectivity with hubs within the other band. When *k* = 30 for *A^*g*^*_VLF_ and *k* = 30 for *A^*g*^*_LF_ is selected, rich-club organizations are formed with hub regions. Bold lines indicate connections among rich-club nodes, showing dense interconnections. The numbers correspond to those in Table 1. Anatomical labels were selected using AAL. The abbreviations represent the direction in the brain: A, anterior; P, posterior; L, left; R, right; D, dorsal; V, ventral. **(G)** Hub regions identified within one wide frequency band (0.01–0.10 Hz) and five narrow frequency bands (0.01–0.03 [VLF], 0.03–0.05, 0.05–0.07, 0.07–0.09 [LF], and 0.09–0.11 Hz). All hub nodes are represented with large green colored spheres. The attributes of the lines are the same as described above.

Anatomical perspectives of hub regions in both networks are shown in Figure 7. Although several ROIs have functional connectivity with hub regions in each network, among the hubs there were dense interconnections or rich-club connections. This finding demonstrates that although there were no significant differences regarding global efficiency between the two frequency-specific networks, their structures contributing to information integration consisted of distinct sets of functional brain hubs that formed distinct rich-club organizations.

Although hub regions in the VLF mainly contained areas of the anterior cingulate and superior medial frontal cortices, those in the LF consisted of the precuneus cortex and thalamus. In order to investigate whether this difference was specific to the relationship between VLF and LF, we identified hub regions within the typical frequency band used for studies of functional connectivity (0.01–0.10 Hz) and within three frequency bands (bandwidth 0.02 Hz) located within 0.01–0.11 Hz without overlapping with the VLF and the LF (0.03–0.05, 0.05–0.07, and 0.09–0.11 Hz; Figure 7G). Within the 0.01–0.10 Hz band, we observed hub regions located in the anterior and posterior cingulate cortices and the thalamus. This supports the notion that network characteristics of integration in the VLF and LF coexist within the network obtained in the wide frequency band. We found that hub regions identified in the frequency bands higher than 0.05 Hz mainly and consistently included ROIs in the precuneus and thalamus, thus supporting the notion that hub regions in the LF reflect representative integration architecture at this frequency range. By contrast, the VLF was the only frequency band where hubs mainly consisted of the medial frontal regions. In the frequency band between 0.03 and 0.05 Hz, which was located between the VLF and frequency ranges over 0.05 Hz, hub regions were identified in both the medial frontal and parietal regions. The network topology of this frequency band might reflect functional characteristics of both networks estimated in the VLF and LF (Figure 7G).

## Discussion

This study investigated frequency specificity of a functional network architecture contributing to information segregation and integration. By calculating coherence among all pairs of ROIs, we found two frequency bands within the range 0.01–0.10 Hz, VLF and LF, where coherence was higher than other frequency bands (Figure 2). Although graph theoretical metrics showed that the network estimated in the VLF had a higher degree of segregation than that in the LF (Figure 4A), no difference was found regarding indices of integration (Figure 4B). By contrast, both frequency-specific networks could be decomposed into a highly similar set of communities corresponding to three functional brain systems (the DMS, FPS, and COS; Figure 5). This indicates that, although networks in the VLF and LF consisted of the same community sets, these were more segregated in the VLF than in the LF. Furthermore, by identifying hub regions in each frequency-specific network, we observed that the hub regions differed in all frequency bands except for one region, the left dorsal anterior precuneus (Table 2); this supported the notion that there were at least two distinct sets of functional hubs depending on the timescale of brain activity. Collectively, our findings demonstrate spontaneous fMRI signal fluctuations in two different frequency bands organized into large-scale networks with distinct topologies for information segregation and integration.

Spontaneous hemodynamic signals include not only fluctuations in spontaneous neural activity but also those generated by physiological signals such as respiratory and cardiac pulsations. Using NIRS has demonstrated that respiratory and cardiac pulsations have typical frequencies (0.3 and 1 Hz), and dominate these frequency bands in the power spectrum (Obrig et al., 2000). Importantly, two additional frequency bands corresponding to the VLF and LF (0.01–0.03 Hz and 0.06–0.08 Hz) have been demonstrated, for which coherence values estimated by signal fluctuations of oxygenated hemoglobin concentration among some distant brain regions are higher than those values in other frequency regions within 0.01–0.10 Hz (Sasai et al., 2011). We obtained a similar result in the current study using both NIRS signals of oxygenated and deoxygenated hemoglobin concentration changes and simultaneously obtained fMRI data analyzed (Figure 3). These results demonstrate that high coherence in the VLF and LF is not due to aliasing of physiological signals in higher frequency regions, and high coherence in the VLF and LF can be consistently observed by different measurement modalities of hemodynamic signals—that is, fMRI and NIRS. Moreover, we conducted additional spectrum analyses on public resting state fMRI data sets and ensured that these characteristics of coherence spectra are universal features of resting state fMRI signals (Figure S1). These results support the hypothesis that high coherences in the VLF and LF of fMRI signals reflect coherent spontaneous neural activities.

In analyzing the brain from the view of graph theory, the minimal node is defined as each voxel in the MRI data set. Although there are studies constructing a voxel-based network of the brain (Eguíluz et al., 2005; Cecchi et al., 2007; van den Heuvel et al., 2008b; Buckner et al., 2009; Hayasaka and Laurienti, 2010), this method is computationally demanding. To reduce the volume of MRI data set, nodes are determined in a larger spatial scale as regions of interest (ROI). ROIs are generally determined by referring the boundary of brain regions that are activated by a specific category of tasks (Dosenbach et al., 2007, 2010; Deshpande et al., 2011; Power et al., 2011; Spreng et al., 2013) or defined as anatomically separate regions (Salvador et al., 2005a,b, 2007, 2008; Achard et al., 2006; Bassett et al., 2008; Hagmann et al., 2008). However, to obtain an accurate description of FCN, ROIs should be selected to represent underlying functional areas of the brain (Butts, 2009; Dosenbach et al., 2010). Therefore, we selected nodes as ROIs defined by a meta-analysis on several task-activation studies in order to ensure reliability on the functional uniformity of voxels within ROIs (Dosenbach et al., 2010). The variability of selections of ROIs can cause inconsistency on results of the network topology. However, hubs and rich-clubs identified in the present study (0.01–0.10 Hz; see Table 2) are located in the similar brain regions that have been repeatedly reported by previous studies using different ROI definitions (see Achard et al., 2006; van den Heuvel et al., 2008b; Buckner et al., 2009; Tomasi and Volkow, 2011a,b; Spreng et al., 2013; for hub; see van den Heuvel and Sporns, 2011 for rich-club). We observed a consistent community assignment with those reported in other studies using distinct ROI sets (Dosenbach et al., 2007; Power et al., 2011; Spreng et al., 2013) as well as a study using the same ROIs (Dosenbach et al., 2010). Thus, it is likely that all of our findings didn't depend on our selection of ROI.

There are several methods for detecting network structures such as communities and hubs; thus, different results might be obtained depending on the method selected. There are also several community detection algorithms for use within a network. However, the optimal algorithm can be selected using a measure of modularity, which has been used as an evaluation function (Newman, 2004, 2006). The algorithm used in the current study was selected because it provided the highest modularity among a given set. Once the optimal algorithm had been selected, the modularity was used as an index to quantify the degree of segregation between detected communities. Using the optimal algorithm for detecting communities in two networks that consist of the same node set, but are connected by two different connection patterns, we could measure and compare how clearly the community structures were present in both networks. In the current study, the same communities were found, but there was a significant difference in modularity between frequency-specific networks in the VLF and LF. These results clearly demonstrate that separation between communities in the network in the VLF was significantly stronger than that in the LF.

The observed hubs were not sensitive to the algorithm selection in the current study. We identified hub regions using two graph measures that characterize different aspects of nodes in a network: the nodal degree and the eigenvector centrality. The nodal degree was defined as the number of connectivity in the current study, and the eigenvector centrality quantified the influence of a node in a network. Individual application of the two measures could potentially detect different hubs in the same network. We therefore defined hubs using both criteria, meaning that our findings were not sensitive to the selection process. We found hub nodes in both networks in the VLF and LF, suggesting that these frequency-specific networks consistently show network structures in which a small set of highly connected and highly influential nodes was present.

“Rich-club” organization is a network structure characterized by the presence of highly interconnected hubs in a network. It was recently demonstrated that rich-club organization is present in structural networks of the nervous system of humans, monkeys, cats, and *C. elegans* (van den Heuvel and Sporns, 2011; Harriger et al., 2012; Towlson et al., 2013; de Reus and van den Heuvel, 2013). Connections within the rich-club organization, which are referred to as “rich-club connections” in the present work, are thought not only to increase the efficiency of global functional integration by bypassing hubs, but also to make the network robust against attacks on the hubs (van den Heuvel and Sporns, 2011). Our findings showing that all of the detected hubs formed rich-club organizations in the current study supports the idea that, independent of frequency-bands, the FCN has a higher-order integration structure that might play a role in the functional integration of the network. Although spatial correspondence among regions of interest are obscure, parts of observed rich-club regions such as the superior frontal, precuneus and thalamus (Figures 7C–F) appear to be included in the structural network reported by van den Heuvel and Sporns (2011). Notably, the rich-club regions in FCNs differed in a frequency-dependent manner within the FCNs of the VLF (Figures 7C,D) and LF (Figures 7E,F). Considering the fact that frequency-specific networks in the VLF and LF have different connection patterns, these diverse rich-club organizations might serve in global, but differential, functional integration specific to the timescales of brain activity.

Using a participation coefficient allowed us to estimate and classify the functional role of hubs in relationship to a community. This measure revealed that all of the hubs in the current study could be classified as “connectors” (Figures 6G,H, and Table 2); this suggests that hubs promote functional integration among the COS, FPS, and DMS in both the VLF and LF. All of the frequency-specific hubs in the VLF were located within the COS, suggesting that it might act as an integration center among these three functional systems in the VLF. By contrast, frequency-specific hubs in the LF were detected in all functional systems (the COS, FPS, and DMS), suggesting that there is no such integration center in the FCN of the LF. Conversely, functional integration might occur directly among all communities within this frequency band. Furthermore, modularity in the LF was significantly lower than that in the VLF—that is, there was more connectivity across boundaries of communities in the FCN for the LF. This supports the idea that direct functional integration among communities can occur more easily in the LF than in the VLF through distributed hubs over all communities and direct connectivity among communities.

The relationship between frequency-components of the fMRI signal and its function has recently been investigated. Some studies focused on the band-specific power of the fMRI signal. For example, Baria et al. (2011) divided fMRI signals into four separate frequency-bands (0.01–0.05, 0.05–0.10, 0.10–0.15, and 0.15–0.20 Hz) and found opposite task-induced shifts in the mean of the whole-brain power between the lowest (0.01–0.05 Hz) and second lowest bands (0.05–0.10 Hz). Other groups decomposed the frequency-band into 0.01–0.027 and 0.027–0.08 Hz and reported frequency-specific relationships of band-limited amplitudes with personality traits (Wei et al., 2014), or among subjects with brain disorders, including amnestic mild cognitive impairment (Han et al., 2011) and schizophrenia (Yu et al., 2014). While these studies focused on the relationship between amplitudes of fMRI signals and functions, Lohmann et al. (2010) found that some regions, including the precuneus and thalamus, show frequency-specific changes of voxel-wise eigenvector centrality between states of hunger and satiety. Furthermore, our findings demonstrated that there are frequency-specific network organizations with distinct topologies in the FCN. Since our data sets were obtained during a resting state, we cannot interpret our results in terms of tasks or behaviors. However, we speculate that differences between network topologies reflect frequency-specific dominance of functional segregation and integration. Although this proposal remains speculative, future investigations exploring the frequency-specific changes of network topology with several tasks and brain states would provide important clues for clarifying the spectral properties of brain functions.

There are two limitations of the methods used in our study. First, non-neural fluctuations included in hemodynamic signals might have affected the current results. It has been demonstrated that hemodynamic signals include signal fluctuations with non-neural physiological origins such as respiratory and cardiac pulsations (Lowe et al., 1998; Bhattacharyya and Lowe, 2004), changes in respiratory and cardiac rates (Wise et al., 2004; Birn et al., 2006; Shmueli et al., 2007), blood pressure (Katura et al., 2006), and changes in vascular tone for cerebral autoregulation (Lagopoulos et al., 2006), and vasomotion (Aalkjaer et al., 2011). Coherence due to the respiratory and cardiac pulsations had peaks out of 0.01–0.10 Hz ([respiratory: ~0.3 Hz] and [cardiac: ~1 Hz]) (Figures 3D,E). Because the sampling rate of fMRI is not sufficiently high, the functional connectivity can be biased by aliasing of these confounds with higher frequencies. By contrast, using NIRS with a sampling rate of 10 Hz revealed that frequency characteristics of functional connectivity are not due to aliasing of noise. However, the frequency ranges of some of these non-neural fluctuations, such as changes in respiratory and cardiac rates, blood pressure, and vasomotion, were included in the hemodynamic signals fluctuating within 0.01–0.10 Hz. Moreover, it is possible that signal fluctuations with non-neural physiological origins are contained uniformly in gray matter voxels (Desjardins et al., 2001; Greicius et al., 2003; Macey et al., 2004). Bias in the estimation of functional connectivity due to physiological confounds can be avoided by using methods that simultaneously record physiological data with fMRI signals (Glover et al., 2000) and/or by exploiting inherent information in the resting state fMRI data (Fox et al., 2005; Chang and Glover, 2009; Anderson et al., 2011; Chai et al., 2012). In the current study, we applied the PSTCor method, which uses only resting state fMRI data sets for correction, in order to factor out non-neural fluctuations to increase confidence in the results of functional connectivity (Anderson et al., 2011). However, although using this method for physiological correction can suppress over-estimation of functional connectivity, it might cause under-estimation because variations in physiological regulations might be correlated with neural activities (Murphy et al., 2013). For example, emotional arousal and activity levels of the autonomic nervous system are indicated by variability of heart rate (Macefield, 2009). Furthermore, it is unclear whether there are regional specific physiological confounds. Because the above-mentioned correction cannot account for all such signals, other methods will be required. Moreover, in comparison to other physiological confounds, the contribution of vasomotion to hemodynamic signals is poorly understood (Murphy et al., 2013). For example, it is still unclear whether vasomotion has an effect on hemodynamics independently of other physiological origins (Morita-Tsuzuki et al., 1992; Hudetz et al., 1998; Biswal and Kannurpatti, 2009). Further studies regarding the relationship between physiology and hemodynamic signals are needed to ensure certainty of results concerning functional connectivity.

Second, state-dependency of functional connectivity must be considered. Although most studies of functional connectivity collect 5–11 min of resting state fMRI data, some have reported non-stationary characteristics using similar length resting state data sets (Chang and Glover, 2010; Hutchison et al., 2012). To characterize the length of time required to acquire reproducible functional connectivity measurements, much effort has been focused on test–retest reliability of functional connectivity maps. Earlier studies showed that the strength of functional connectivity becomes stable when using data sets longer than 7 min (Shehzad et al., 2009; Van Dijk et al., 2010; Thomason et al., 2011; Braun et al., 2012; Li et al., 2012). However, a recent investigation into this issue with a longer scan length (27 min) than that in previous studies demonstrated that improvements in test–retest reliability plateaued around 12–16 min for intra-session comparisons and at 8–12 min for inter-session comparisons (Birn et al., 2013). Therefore, to achieve reliable results, we need to acquire resting state data for longer than 16 min. Because we used 20 min resting state data to calculate functional connectivity in the current study, we were able to derive our results free from this bias. However, it should be noted that although the highest test–retest reliabilities could be provided by the scan length used in the current study, the degree of reliability for inter-session variability is lower than that of intra-session variability (Birn et al., 2013). Further investigation into the state-dependency of the FCN would provide further insights.

Theoretical views on the anatomical wiring of the brain suggest that it has a fundamental characteristic of reconciling the apparently opposing demands of local segregation and global integration of information (Felleman and Van Essen, 1991; Tononi et al., 1994). This attribute has also been demonstrated to exist in the network structure of functional brain organization (Sporns, 2013). Our current results suggest that the spatial order of the brain can vary by measuring it with different timescales. Indeed, we demonstrated the co-existence of two functional brain organizations in a frequency-dependent manner. Since different network topologies might contribute to different brain functions, the present study promotes further investigation into the relationship between frequency-specific network topologies and the timescales of human behavior.

### Conflict of interest statement

The authors declare that the research was conducted in the absence of any commercial or financial relationships that could be construed as a potential conflict of interest.
