# Supplementary figures and images for: Frequency-specific network topologies in the resting human brain
Source: Front Hum Neurosci. 2014 Dec 22;8:1022. doi: 10.3389/fnhum.2014.01022 (PMC4273625; doi:10.3389/fnhum.2014.01022)

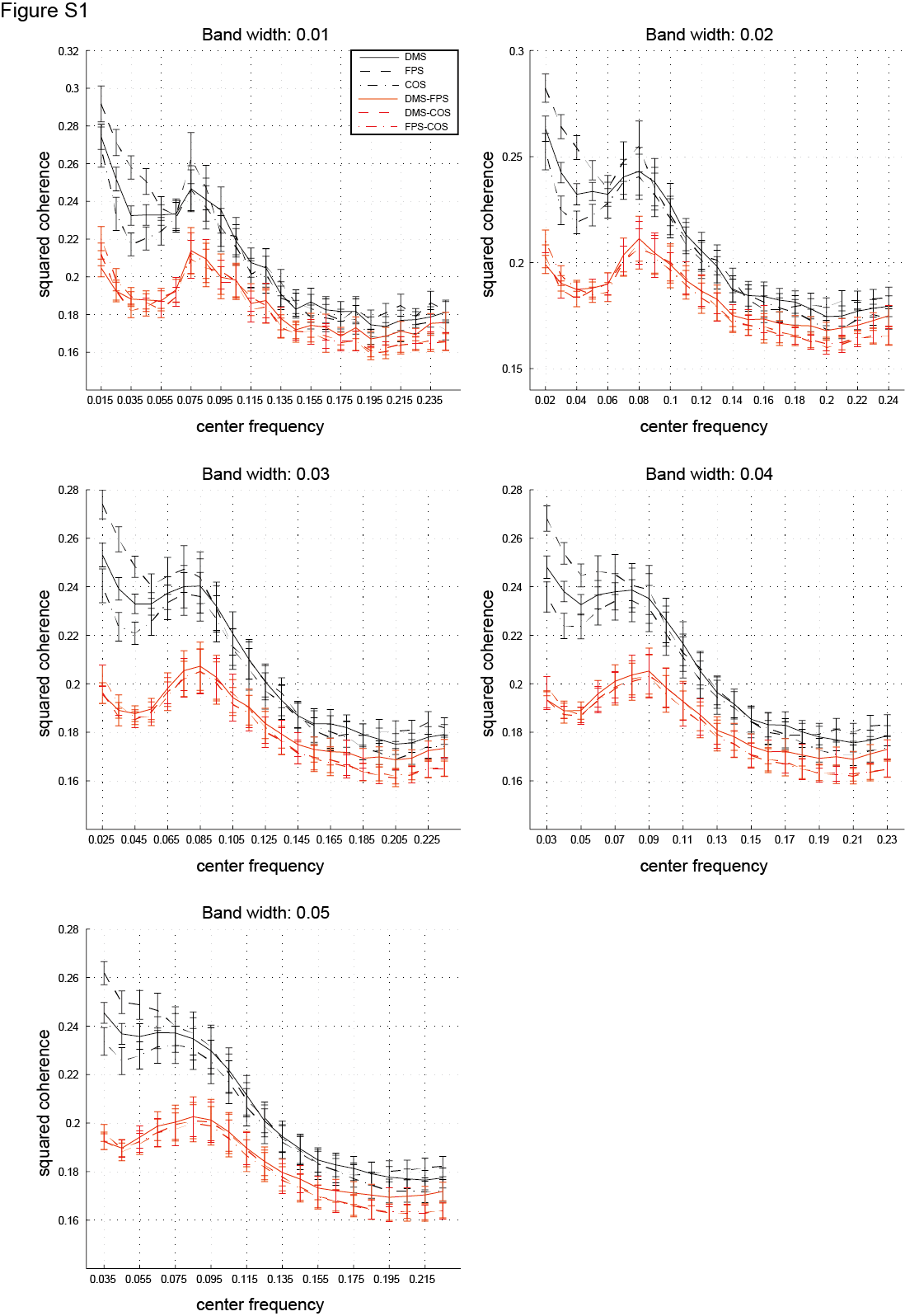

Supplement: Supplementary file 1 [file Image1.PNG]

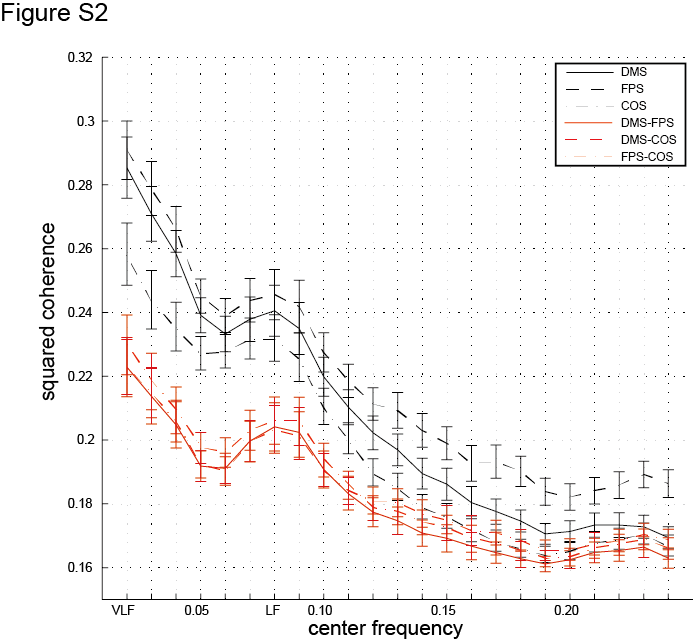

Supplement: Supplementary file 2 [file Image2.PNG]
